# Placenta Prævia

**Published:** 1883-02

**Authors:** 


Placenta Prævia.—Dr. John Reid reports a case of placenta prævia, in the *British Medical Journal,* which terminated rather differently from what is generally supposed to be the rule. Having intentionally lacerated the placenta to some extent, with the result of but covering his fingers with blood, he ruptured the membraues, when only some blood-streaked liquor amnii escaped. Within three hours the labor terminated naturally. The placenta was soon expelled, but the uterus still felt like a large doughy mass; but soon after the expulsion of about a pint of blood, firm contraction of the uterus resulted, and the case progressed favorably. This is one of the many cases which militate against Simpson's theory of hemorrhage in placenta prævia, and may also add some additional force to the objections against meddlesome midwifery.—*Medical and Surgical Reporter.*

